# AT1R in Central Nervous System Disorders: Unveiling Novel Mechanisms and Therapeutic Avenues for Addiction

**DOI:** 10.1111/adb.70152

**Published:** 2026-04-14

**Authors:** Jianan Lv, Dan Zhu, Sisi Song, Jiahui Zhou, Xiaoyu Zhang, Wenhua Zhou, Yu Liu, Zizhen Si

**Affiliations:** ^1^ School of Pharmacy, Health Science Center Ningbo University Ningbo China; ^2^ School of Basic Medical Sciences, Health Science Center Ningbo University Ningbo China; ^3^ Department of Psychology, Collage of Teacher Education Ningbo University Ningbo China; ^4^ Zhejiang Provincial Key Laboratory of Addiction Research The Affiliated Kangning Hospital of Ningbo University Ningbo China

**Keywords:** angiotensin II type 1 receptor, neuropsychiatric disorders, psychostimulants, substance use disorders, therapeutic target

## Abstract

Although the angiotensin II type 1 receptor (AT1R), a pivotal component of the renin–angiotensin system (RAS), is associated with cardiovascular and renal homeostasis, burgeoning evidence implicates its critical role in neuropsychiatric disorders, particularly addiction. Beyond regulating haemodynamics, AT1R activation in the central nervous system (CNS) modulates neuroinflammatory cascades, dopaminergic signalling plasticity, and stress‐responsive neural circuit processes central to addiction pathophysiology. Notably, preclinical studies reveal that AT1R blockade attenuates drug‐seeking behaviours by normalizing mesolimbic dopamine dysregulation and reducing glutamatergic excitotoxicity in the nucleus accumbens. This review systematically integrates contemporary evidence elucidating the dual pathophysiological roles of AT1R in CNS disorders, with particular emphasis on neurodegenerative diseases and psychiatric conditions. Crucially, we delineate two mechanistically distinct yet interconnected functions of AT1R: (1) serving as a critical mediator of maladaptive neuroplasticity during protracted exposure to addictive substances and (2) functioning as a regulator of blood–brain barrier (BBB) integrity, thereby potentiating neurotoxicant infiltration in substance use disorders. Building upon these mechanistic insights, we propose a translational framework for repurposing clinically approved AT1R antagonists as novel pharmacotherapies targeting addiction‐related neurocircuitry dysregulation. By bridging molecular insights with translational opportunities, this work positions AT1R as a novel therapeutic target to address unmet clinical needs in addiction.

## Graphical abstract

AT1R signaling drives addiction through interconnected pathways, and AT1R antagonists target multiple nodes for therapeutic effects.

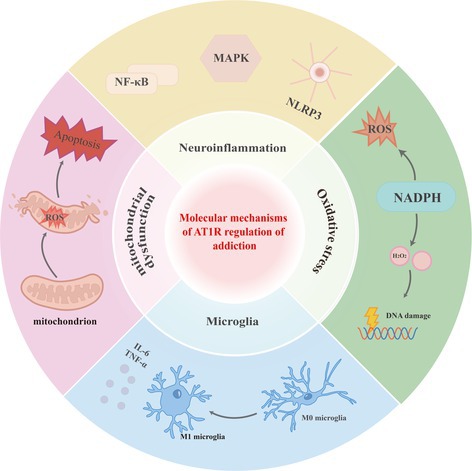

## 1 Introduction

### 1.1 The Renin–Angiotensin System and the Central Nervous System: An Evolving Crossroad

The renin–angiotensin system (RAS), traditionally recognized for its peripheral role in blood pressure regulation and fluid homeostasis, has emerged as a critical neuromodulatory network within the central nervous system (CNS) [1]. Angiotensin II (Ang II), the primary effector peptide of RAS, is synthesized both peripherally and within the brain [2, 3]. Within the CNS, Ang II generation arises from both peripheral influx and local synthesis. Peripherally derived Ang II accesses brain regions lacking a complete blood–brain barrier (BBB), primarily the circumventricular organs (CVOs; e.g., subfornical organ and area postrema) [4]. Locally synthesized Ang II occurs in nuclei with intact BBB (e.g., hypothalamic paraventricular nucleus and brainstem nucleus tractus solitarius) via tissue‐specific enzymatic pathways, independent of renal renin [5]. Beyond these regions, astrocyte‐derived angiotensinogen contributes to parenchymal Ang II synthesis, enabling autocrine/paracrine signalling [6]. Ang II exerts its effects via two primary G protein‐coupled receptors: angiotensin II type 1 receptor (AT1R) and type 2 receptor (AT2R). AT1R is widely distributed in brain regions critical for autonomic and behavioural regulation, such as the hypothalamus, basal ganglia, prefrontal cortex and mesolimbic dopamine pathways—key circuits implicated in reward processing and addiction [7]. In contrast, research on AT2R is relatively limited, and AT2R exhibits a more restricted expression pattern, predominantly localized to sensory and motor nuclei during development [8]. Functionally, AT1R activation triggers canonical pathways (e.g., Gq/11‐mediated phospholipase C activation, Ca^2+^ mobilization) and non‐canonical signalling (e.g., β‐arrestin‐dependent MAPK/ERK cascades), driving pro‐inflammatory and pro‐oxidant responses [9, 10, 11]. Conversely, AT2R engages Gi/o‐coupled pathways, counteracting AT1R effects by promoting neuroprotection, synaptic plasticity and anti‐inflammatory responses through nitric oxide (NO) synthesis and protein phosphatase 2A (PP2A) activation [12, 13]. This receptor dichotomy positions the brain RAS as a dynamic rheostat, balancing neuroadaptation and maladaptive plasticity in CNS disorders.

**FIGURE 1:**
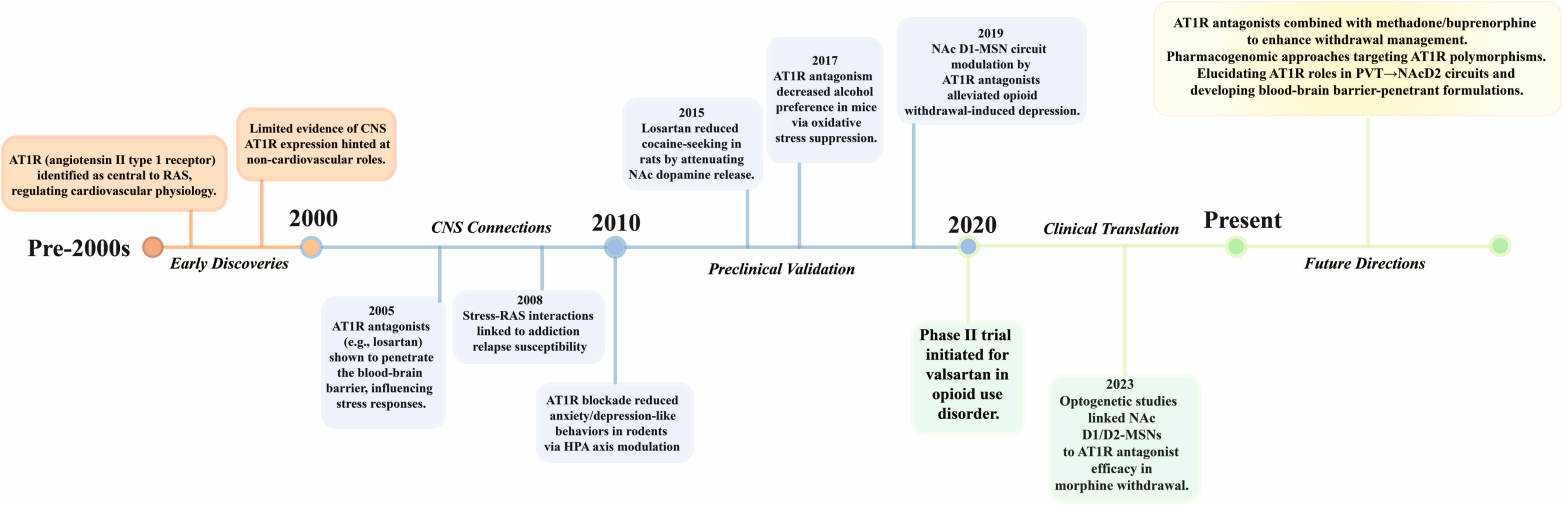
Chronological research timeline: AT1R antagonists and drug addiction.

The functional interplay between Ang II receptors extends beyond cardiovascular regulation to modulate neuroendocrine, cognitive and reward‐related behaviours. AT1R activation in the paraventricular nucleus stimulates corticotropin‐releasing hormone (CRH) release, linking stress axis hyperactivity to addiction vulnerability [7]. In the ventral tegmental area (VTA), a dysregulated RAS contributes to alcohol addiction by stimulating oxidative stress and dopaminergic neurotransmission [14]. AT2R activation in the prefrontal cortex dampens stress‐induced reinstatement of drug‐seeking behaviour by normalizing hypoactive glutamatergic transmission [15]. Notably, brain RAS exhibits region and cell type‐specificity: microglial AT1R drives neuroinflammation via NADPH oxidase‐derived ROS activation, while neuronal AT2R stimulation enhances axonal plasticity after spinal cord injury via upregulating BDNF expression [16, 17]. Emerging evidence also highlights the role of alternative RAS components (e.g., angiotensin‐(1–7)/Mas receptor axis) in mitigating AT1R‐mediated neurotoxicity, suggesting a multilayered regulatory network. Dysregulation of this balance is implicated in diverse CNS pathologies, from hypertension‐associated cognitive decline to methamphetamine‐induced hyperlocomotion and neurotoxicity [18, 19]. These findings underscore the CNS RAS as a pleiotropic modulator of disease trajectories, with AT1R emerging as a pivotal target for addiction therapeutics due to its convergence on both reward circuitry and stress‐adaptive mechanisms (Figure 1).

### 1.2 Biological Properties and Signalling Pathways of AT1R

AT1R is a member of the GPCR family and consists of seven transmembrane domains with an extracellular N‐terminus and an intracellular C‐terminus [20]. Its activation is primarily triggered by the binding of Ang II, which induces a conformational change in the receptor, leading to the dissociation of the G protein into its α and βγ subunits and facilitating its coupling to heterotrimeric G proteins, predominantly Gq/11 [21, 22]. This interaction initiates downstream signalling cascades. Activation of AT1R elicits a complex network of intracellular signalling pathways, many of which are implicated in disease pathogenesis, including the generation of reactive oxygen species (ROS), activation of nuclear factor‐kappa B (NF‐κB) and stimulation of mitogen‐activated protein kinase (MAPK) pathways [23]. These signalling pathways are crucial for mediating the physiological and pathological effects of AT1R.

**FIGURE 2:**
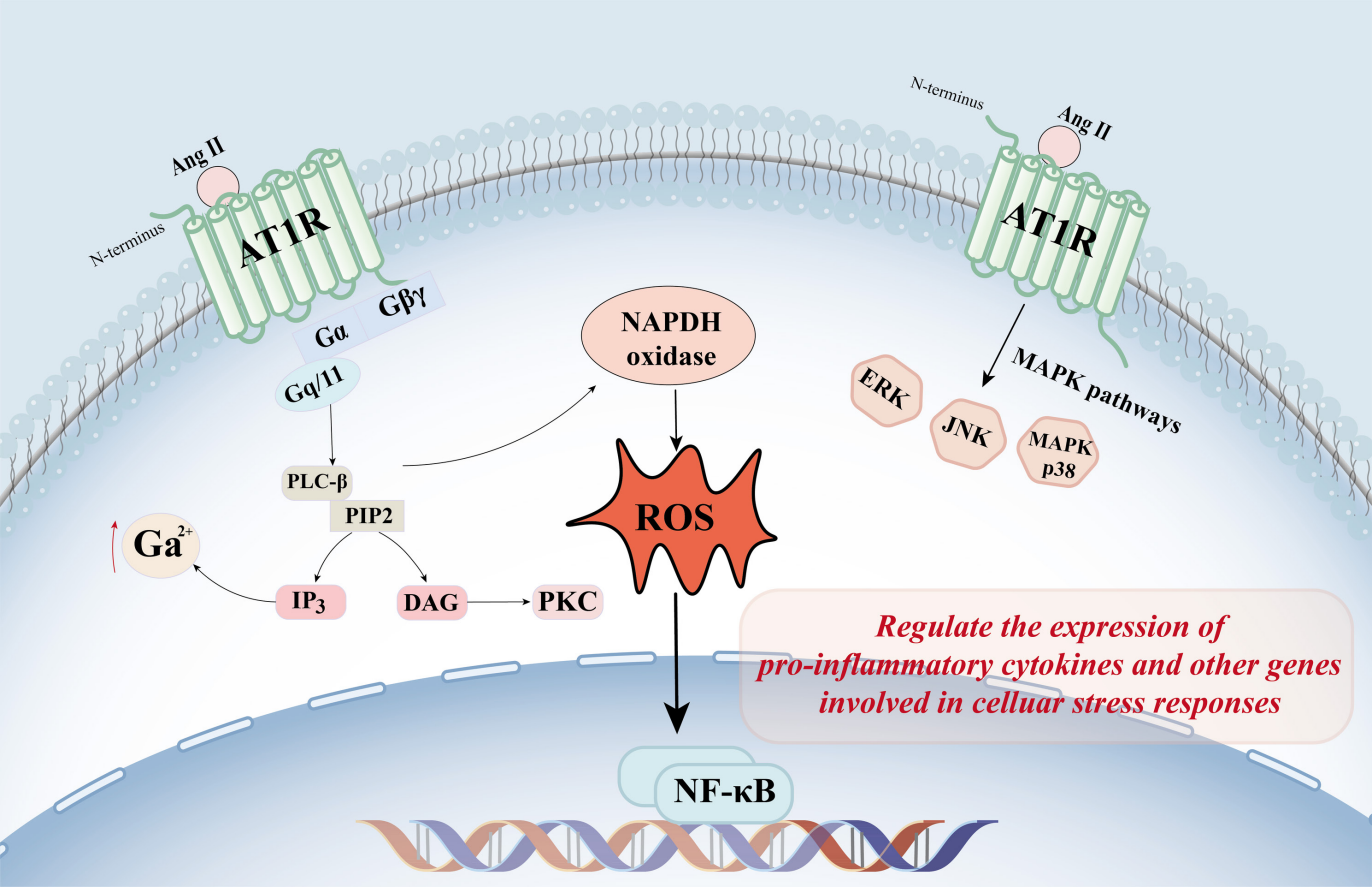
The signalling pathways of AT1R. Upon activation, the AT1R predominantly couples with the Gq/11 protein, thereby stimulating PLC to hydrolyze PIP2 into IP3 and DAG. IP3 triggers the release of calcium from intracellular stores, whereas DAG activates PKC. These signalling events subsequently lead to the generation of ROS via the activation of NADPH oxidase, a pivotal enzyme implicated in oxidative stress. ROS can further activate NF‐κB, a transcription factor that modulates the expression of pro‐inflammatory cytokines and other genes associated with cellular stress responses. Moreover, AT1 receptor activation also stimulates the MAPK pathway, encompassing ERK, JNK and p38 MAPK.

Upon activation, AT1R primarily couples to the Gq/11 protein, which stimulates phospholipase C (PLC) to hydrolyze phosphatidylinositol 4,5‐bisphosphate (PIP2) into inositol trisphosphate (IP3) and diacylglycerol (DAG). IP3 induces the release of calcium from intracellular stores, while DAG activates protein kinase C (PKC) [10]. These events lead to the generation of ROS through the activation of NADPH oxidase, a key enzyme in oxidative stress. ROS, in turn, can activate NF‐κB, a transcription factor that regulates the expression of pro‐inflammatory cytokines and other genes involved in cellular stress responses [24]. Additionally, AT1R activation stimulates the MAPK pathway, including extracellular signal‐regulated kinase (ERK), c‐Jun N‐terminal kinase (JNK) and p38 MAPK. These kinases regulate various cellular processes such as proliferation, differentiation and apoptosis [25]. The interplay between these signalling pathways underscores the complexity of AT1R's role in cellular physiology (Figure 2).

### 1.3 The Neurobiological Basis of Drug Addiction

Drug addiction is a chronic, relapsing disorder characterized by compulsive drug seeking and use despite adverse consequences [26]. Its neurobiological basis lies in the profound alterations in brain circuits involved in reward, motivation, memory and executive control. Central to these circuits are the mesolimbic and mesocortical dopamine pathways, which originate in the ventral tegmental area (VTA) and project to the nucleus accumbens (NAc), prefrontal cortex (PFC), amygdala (AMY) and hippocampus (HP) [27]. The NAc, particularly its shell region, is a critical hub for reward processing and reinforcement learning. It integrates dopaminergic inputs from the VTA with glutamatergic inputs from the PFC, AMY and HP, enabling the association of drug‐related cues with rewarding outcomes [28]. The PFC, especially the dorsolateral and orbitofrontal regions, plays a pivotal role in decision‐making, impulse control and the regulation of drug‐seeking behaviour. Dysfunction in the PFC, often observed in addiction, leads to impaired inhibitory control and heightened sensitivity to drug‐associated stimuli [29]. Additionally, the AMY contributes to the emotional salience of drug cues while the HP is involved in the formation and retrieval of drug‐related memories. Together, these brain regions form a network that underlies the transition from voluntary drug use to compulsive addiction [30].

Drugs of abuse, such as cocaine, opioids and amphetamines, directly or indirectly increase dopamine levels in the NAc by enhancing dopamine release, blocking reuptake or stimulating dopamine receptors [27]. This surge in dopamine signalling reinforces drug‐taking behaviour by activating reward‐related circuits. Chronic drug exposure, however, leads to neuroadaptations that disrupt normal dopaminergic function [31]. For instance, prolonged dopamine elevation results in the downregulation of dopamine D2 receptors and reduced dopamine synthesis and release, a phenomenon known as hypodopaminergia. This blunted dopamine response diminishes the brain's sensitivity to natural rewards, creating a state of anhedonia and driving individuals to seek higher drug doses to achieve the same pleasurable effects [32]. Furthermore, chronic drug use induces glutamatergic plasticity in the PFC‐NAc pathway, strengthening drug‐related memories and weakening cognitive control over drug‐seeking behaviour. These neuroadaptations contribute to the persistence of addiction and the high risk of relapse, even after prolonged abstinence [33]. Understanding the interplay between dopaminergic signalling and addiction‐related brain regions provides critical insights into the mechanisms underlying this disorder and highlights potential targets for therapeutic intervention. The transition from recreational drug use to addiction involves a shift from dopamine‐driven reward processing to dysregulated motivational and cognitive control mechanisms. Initially, drugs of abuse hijack the brain's natural reward system by producing a rapid and intense dopamine release in the nucleus accumbens, creating a powerful reinforcement signal. This acute dopamine surge activates D1 receptors, which strengthen synaptic connections in the NAc and other reward‐related regions through mechanisms such as long‐term potentiation (LTP) [34]. Over time, however, repeated drug exposure leads to neuroadaptive changes that alter the balance between dopamine and other neurotransmitter systems [35]. For example, the downregulation of D2 receptors in the striatum and PFC reduces the brain's ability to experience pleasure from natural rewards, whereas the upregulation of D1 receptors enhances the salience of drug‐related cues. This imbalance promotes compulsive drug‐seeking behaviour, as individuals become increasingly driven by the need to alleviate withdrawal symptoms and restore dopamine levels [36].

**FIGURE 3:**
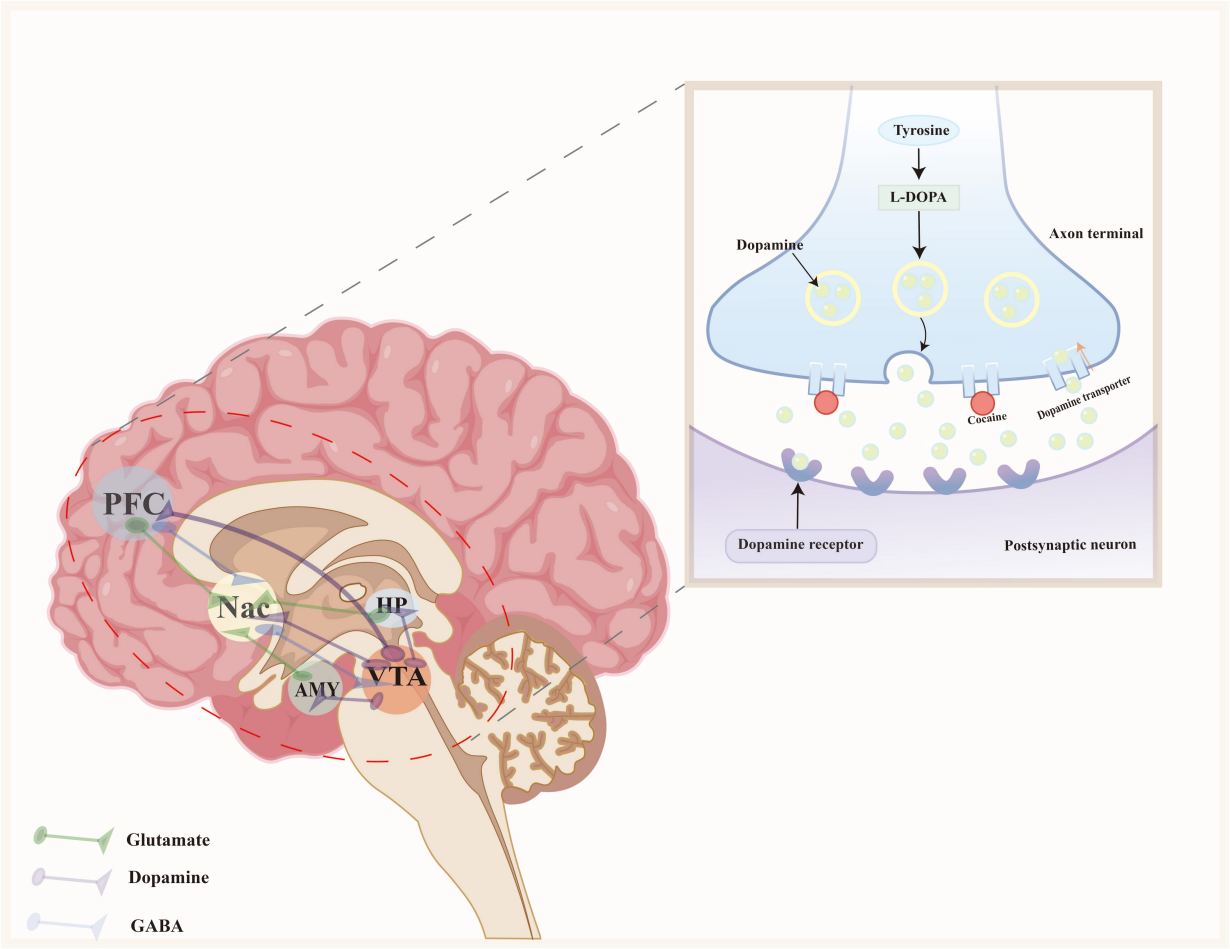
The reward circuit of the brain. Ventral tegmental area (VTA); nucleus accumbens (NAc); prefrontal cortex (PFC); amygdala (AMY); hippocampus (HP). The brain's reward circuit is a network of interconnected regions that mediate pleasure, motivation, learning and reinforcement of behaviours. It is primarily driven by dopamine (DA) signalling but also involves other neurotransmitters like GABA and glutamate. The reward circuit is a dopamine‐centred network that evolved to reinforce survival behaviours but is vulnerable to hijacking by addictive substances. It integrates motivation (‘wanting’), pleasure (‘liking’), learning (‘memory’) and habit formation, with disruptions leading to addiction, depression and impulse disorders. Then, cocaine binds to dopamine transporters, thereby inhibiting dopamine reuptake and maintaining neurons in a state of sustained excitation.

In addition to dopaminergic dysregulation, addiction is characterized by maladaptive changes in glutamatergic and GABAergic signalling [37]. Chronic drug use disrupts the normal functioning of the PFC, impairing its ability to exert top‐down control over subcortical regions such as the NAc and AMY. This loss of inhibitory control is exacerbated by drug‐induced alterations in GABAergic interneurons, which further disinhibit glutamatergic projections to the NAc [38]. The resulting hyperglutamatergic state strengthens drug‐related memories and enhances cue‐induced craving, making it difficult for individuals to resist relapse. Moreover, stress systems, particularly the hypothalamic–pituitary–adrenal (HPA) axis and corticotropin‐releasing factor (CRF) signalling, become dysregulated in addiction, further amplifying the drive to seek drugs [39]. These neurobiological changes create a vicious cycle of craving, drug use and withdrawal, perpetuating the addictive state. By elucidating the complex interplay between dopaminergic, glutamatergic and stress systems, researchers can identify novel therapeutic strategies to restore normal brain function and improve outcomes for individuals struggling with addiction (Figure 3).

## 2 The Dual Role of AT1R in CNS Diseases

### 2.1 Neurodegenerative Disease

AT1R has emerged as a critical player in the pathophysiology of neurodegenerative diseases such as Alzheimer's disease (ad) and Parkinson's disease (PD). In these conditions, overactivation of AT1R contributes to neuroinflammation and oxidative stress, which exacerbate neuronal damage and disease progression [40, 41]. AT1R is widely expressed in the brain, particularly in regions vulnerable to neurodegeneration, such as the hippocampus, substantia nigra and cortex [7]. Under pathological conditions, excessive activation of AT1R by its ligand Ang II triggers the production of ROS through the activation of NADPH oxidase, which is directly stimulated by AT1R through the Gq/11‐PLC‐PKC pathway [42]. NADPH oxidase generates superoxide anions, which react with nitric oxide (NO) to form peroxynitrite, a highly reactive nitrogen species that causes nitrosative stress and further damages neurons [43]. Additionally, ROS activate redox‐sensitive transcription factors, including NF‐κB, which promotes the expression of pro‐inflammatory cytokines such as interleukin‐6 (IL‐6) and tumour necrosis factor‐alpha (TNF‐α) [44]. These cytokines, in turn, amplify neuroinflammatory responses by activating microglia and astrocytes, leading to a self‐perpetuating cycle of inflammation and oxidative stress. Furthermore, AT1R activation inhibits the nuclear factor erythroid 2‐related factor 2 (Nrf2) pathway, a key antioxidant defence mechanism, further tipping the balance towards oxidative damage [45]. In ad, AT1R overactivation has been linked to the accumulation of amyloid‐beta (Aβ) plaques and tau hyperphosphorylation, both of which are exacerbated by oxidative stress and neuroinflammation [46]. Similarly, in PD, AT1R‐mediated oxidative stress contributes to the degeneration of dopaminergic neurons in the substantia nigra, a hallmark of the disease [47]. Thus, AT1R overactivation serves as a critical nexus between neuroinflammation, oxidative stress and neurodegeneration.

### 2.2 Distinctive Mechanisms of Drug Addiction

#### 2.2.1 AT1R With Reward Loops

AT1R is expressed in brain regions central to reward processing [7]. These regions form the core of the mesocorticolimbic dopamine (DA) system, which is pivotal for reward signalling, reinforcement learning and the development of addictive behaviours [36]. Activation of AT1R in these regions has been shown to modulate DA release, a neurotransmitter that plays a central role in the rewarding effects of drugs of abuse [48, 49]. Preclinical studies have demonstrated that pharmacological blockade of AT1R attenuates drug‐induced DA surges in the NAc, suggesting that AT1R signalling is necessary for the full expression of drug reward [50]. Furthermore, AT1R activation influences the expression and function of DA transporters (DAT) and receptors, thereby altering DA signalling dynamics [51]. These findings collectively highlight AT1R as a critical modulator of DA neurotransmission and a potential therapeutic target for mitigating the dysregulation of reward circuitry in addiction (Table 1).

**TABLE 1:** Critical role of AT1R in addiction‐related neural mechanisms.

Pathophysiological domain	Mechanisms	Brain regions/molecules involved	Functional impact	References
Dopaminergic system dysregulation	AT1R activation increases VTA DA neuron firing, amplifying NAc DA release; AT1R antagonism blunts drug‐evoked DA overflow; alters DAT trafficking and D1/D2 receptor sensitivity	VTA, NAc, DA, DAT	Drives reward salience and compulsive drug‐seeking	[50]
Maladaptive synaptic remodelling	AT1R‐NMDAR crosstalk enhances LTP in NAc‐PFC circuits; Ang II/AT1R signalling augments GluN2B phosphorylation in MSNs; upregulates AMPAR surface expression; AT1R blockers normalize spine density	NAc, PFC, NMDAR, AMPAR, GluN2B	Encodes persistent drug–cue associations and habit formation	[52]
Neuroinflammatory priming	AT1R‐dependent microglial activation elevates IL‐6/TNF‐α in mesolimbic regions	NAc, VTA, IL‐6, TNF‐α, CX3CR1	Sustains hyperdopaminergic and hyperglutamatergic states	[53]
Translational pharmacotherapy	Telmisartan reduce cocaine CPP and relapse‐like behaviour in rodents; rescue DA/glutamate homeostasis via dual neural‐immune modulation	Telmisartan, valsartan	Clinically actionable targets for relapse prevention and cognitive recovery	[54]

In addition to its effects on DA release, AT1R plays a significant role in synaptic plasticity, a neural mechanism underlying learning and memory processes that are hijacked in addiction [55, 56]. Chronic drug exposure induces long‐term changes in synaptic strength, particularly in glutamatergic synapses within the mesolimbic pathway [57]. These changes are thought to underlie the persistence of addictive behaviours, including craving and relapse. AT1R activation has been shown to enhance N‐methyl‐D‐aspartate receptor (NMDAR) function, promoting LTP in the NAc, which is associated with the consolidation of drug‐related memories. Ang II, as the primary ligand for AT1R, potentiates NMDAR‐mediated currents in NAc medium spiny neurons, a mechanism that may contribute to the strengthening of drug‐associated synaptic connections [58]. Moreover, AT1R signalling modulates the expression of synaptic proteins, such as postsynaptic density protein 95 (PSD‐95) and α‐amino‐3‐hydroxy‐5‐methyl‐4‐isoxazolepropionic acid receptors (AMPARs), further influencing synaptic plasticity [52]. AT1R blockade has been demonstrated to reverse drug‐induced synaptic adaptations, suggesting its potential in restoring normal synaptic function [59] (Table 1).

The involvement of AT1R in addiction extends beyond its direct effects on DA neurotransmission and synaptic plasticity, encompassing broader neuroinflammatory and neuroendocrine mechanisms that contribute to reward circuit dysfunction. Chronic drug use induces neuroinflammation, characterized by microglial activation and increased cytokine release, which exacerbates the neurobiological alterations associated with addiction [60]. AT1R activation has been implicated in promoting neuroinflammatory responses in reward‐related brain regions, thereby perpetuating the pathological changes observed in addiction. AT1R signalling enhances the production of pro‐inflammatory cytokines, such as IL‐6 and TNF‐α, in the NAc and VTA [53]. These cytokines can dysregulate DA signalling by altering the expression of DA receptors and transporters, as well as impairing synaptic plasticity through mechanisms involving oxidative stress and excitotoxicity. The multifaceted role of AT1R in addiction highlights its potential as a therapeutic target.

#### 2.2.2 Preclinical Evidence

AT1R antagonists, such as losartan, have emerged as promising therapeutic agents for reducing drug‐seeking behaviour in preclinical models of addiction. Accumulating evidence suggests that AT1R blockade attenuates the reinforcing effects of various addictive substances, including cocaine, opioids and alcohol. In rodent models of cocaine addiction, systemic administration of losartan has been shown to significantly reduce both self‐administration and the reinstatement of drug‐seeking behaviour, a hallmark of relapse [54]. These effects are thought to be mediated, at least in part, by the modulation of the mesolimbic dopamine system, which includes the VTA and NAc [61]. This system is central to the brain's reward circuitry and is critically dysregulated by chronic drug exposure. AT1R antagonists appear to restore normal dopamine signalling, thereby reducing the motivational drive to seek drugs. By reducing neuroinflammation and oxidative stress, AT1R antagonists may help to restore neuronal homeostasis and reduce the persistence of addiction‐related behaviours [62]. These findings collectively suggest that AT1R antagonists could offer a novel therapeutic strategy for addiction by targeting both the neurochemical and neuroinflammatory mechanisms underlying the disorder.

The mechanisms by which AT1R antagonists exert their anti‐addictive effects are complex and multifaceted, involving both synaptic and non‐synaptic processes. AT1R antagonists have been shown to reverse the drug‐induced synaptic alterations, potentially through the regulation of glutamate neurotransmission [63]. Glutamate is the primary excitatory neurotransmitter in the brain and plays a central role in synaptic plasticity. Chronic drug exposure disrupts glutamate homeostasis, leading to hyperexcitability and aberrant synaptic remodelling [64]. By restoring glutamate balance, AT1R antagonists may help to normalize synaptic plasticity and reduce the reinforcing effects of drugs. Additionally, AT1R blockade has been found to modulate the expression of stress‐related peptides, such as CRF, in the amygdala. Chronic drug use dysregulates the CRF system, leading to heightened stress responses and increased vulnerability to relapse. By reducing CRF expression and attenuating the stress response, AT1R antagonists may diminish the negative reinforcement that drives continued drug use [65]. AT1R antagonists have been shown to influence the HPA axis, which is often hyperactivated in individuals with substance use disorders. By normalizing HPA axis activity, these drugs may further reduce stress‐induced drug‐seeking behaviour [66]. Importantly, the therapeutic potential of AT1R antagonists is bolstered by their well‐established safety profile, as these drugs are already widely used in the treatment of hypertension and other cardiovascular conditions. This makes them particularly attractive candidates for repurposing in the treatment of addiction. However, despite the encouraging results of the preclinical studies, several questions remain unanswered. The precise molecular and cellular mechanisms by which AT1R antagonists modulate synaptic plasticity and stress responses in the context of addiction require further elucidation. Future research should also explore the effects of AT1R antagonists on other addictive substances, such as nicotine and methamphetamine, to determine the breadth of their therapeutic potential.

## 3 Molecular Mechanisms of AT1R Regulation of Addiction

### 3.1 Neuroinflammation and Glial Cell Activation

**FIGURE 4:**
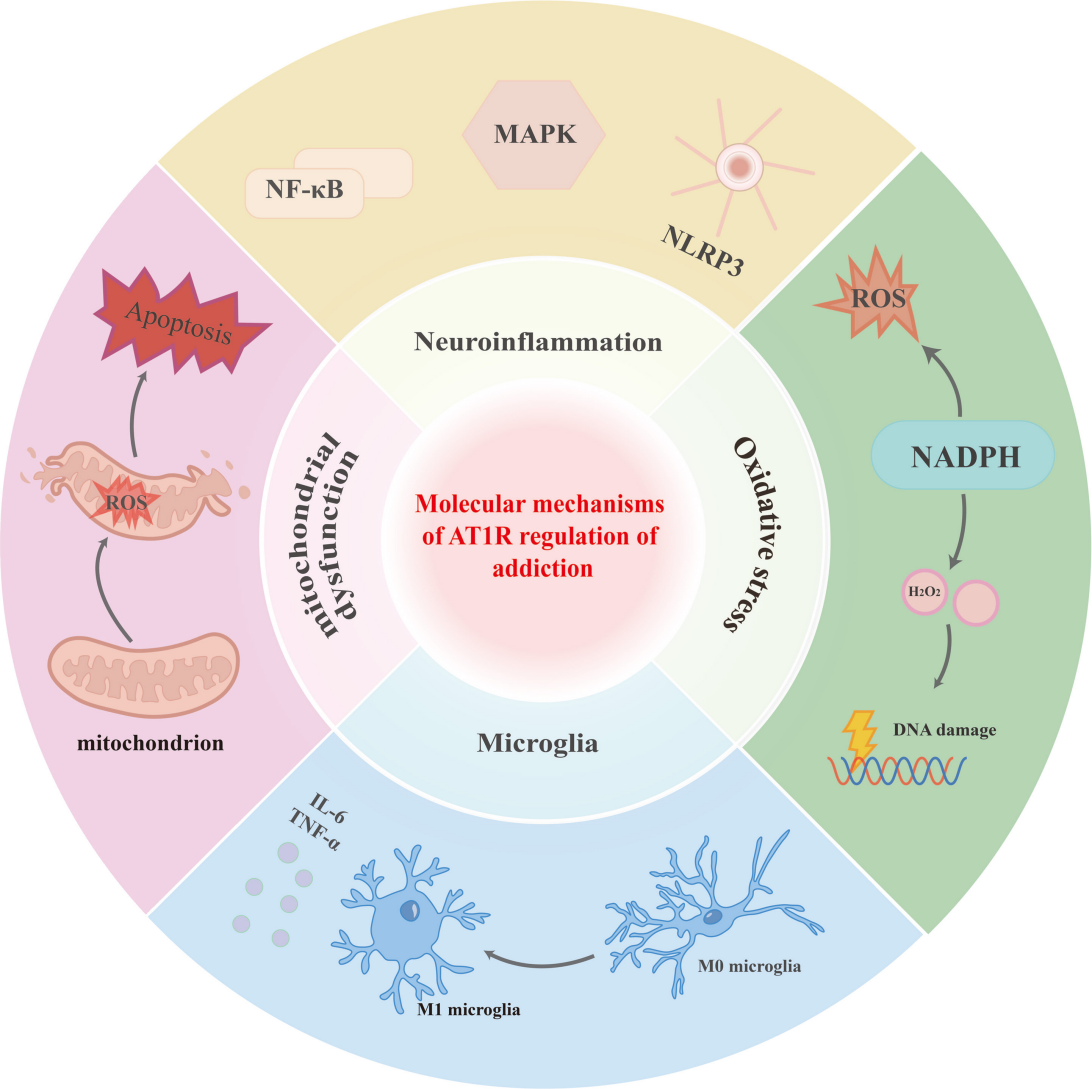
AT1R signalling drives addiction neuropathology through interconnected molecular cascades, and AT1R antagonists exert therapeutic effects by targeting multiple nodes. Chronic drug exposure activates AT1R, triggering downstream pathways that collectively sustain addiction: (1) MAPK pathway → apoptosis and DNA damage; (2) NF‐κB/NLRP3 pathway → neuroinflammation; (3) NADPH oxidase/ROS pathway → mitochondrial dysfunction and oxidative stress; (4) microglial M1 polarization → release of IL‐6/TNF‐α, sensitizing reward circuitry. AT1R antagonists block these cascades at key nodes: they inhibit MAPK‐driven apoptosis, suppress NF‐κB‐mediated inflammation, reduce NADPH oxidase‐derived ROS and prevent microglial M1 polarization. This multitarget intervention normalizes neuronal homeostasis, attenuates drug‐seeking behaviour and reduces relapse vulnerability.

Microglia, the primary immune effector cells of the CNS, express AT1R, and its activation by Ang II initiates a series of intracellular signalling cascades that drive microglial activation [44]. Upon AT1R stimulation, microglia undergo a phenotypic shift from a surveillant state to an activated state, characterized by morphological changes and the upregulation of pro‐inflammatory signalling pathways. Key among these pathways are the NF‐κB and MAPK cascades, which orchestrate the transcriptional activation of genes encoding pro‐inflammatory cytokines, including IL‐6 and TNF‐α [67, 68] (Figure 4). These cytokines are critical mediators of neuroinflammation, contributing to synaptic dysfunction, neuronal apoptosis and the behavioural and cognitive deficits observed in neuropsychiatric disorders such as depression, anxiety and substance use disorders. The AT1R‐mediated activation of microglia and the subsequent release of IL‐6 and TNF‐α create a self‐perpetuating cycle of inflammation, exacerbating neuronal damage and perpetuating the pathological processes underlying these conditions [69]. This inflammatory cascade is not limited to acute responses but can also contribute to chronic neuroinflammation, which is increasingly recognized as a key driver of neuropsychiatric disease progression.

The therapeutic potential of targeting AT1R signalling to mitigate neuroinflammation and its consequences has been demonstrated in preclinical studies. AT1R antagonists have been shown to effectively suppress microglial activation and reduce the production of IL‐6 and TNF‐α in various models of neuropsychiatric disorders. These pharmacological agents exert their effects by blocking AT1R‐mediated signalling pathways, thereby attenuating the activation of NF‐κB and MAPK and reducing the transcription of pro‐inflammatory cytokines [16, 70]. In animal models of depression, AT1R antagonists have been shown to alleviate depressive‐like behaviours and restore synaptic plasticity, likely through their anti‐inflammatory effects [71]. Similarly, in models of substance use disorders, AT1R blockade has been associated with reduced drug‐seeking behaviour and relapse, suggesting that AT1R signalling may modulate reward pathways through its effects on neuroinflammation [50]. Existing studies have found that AT1R antagonists may promote the resolution of inflammation by enhancing the release of anti‐inflammatory cytokines, such as interleukin‐10 (IL‐10), and by facilitating the transition of microglia from a pro‐inflammatory to a reparative phenotype [72, 73]. These findings underscore the potential of AT1R antagonists as a novel therapeutic strategy for neuropsychiatric disorders, particularly those characterized by chronic neuroinflammation. Moreover, although preclinical studies have provided compelling evidence for the efficacy of AT1R antagonists, clinical trials are needed to evaluate their safety and effectiveness in human populations. Future research should also explore the potential synergistic effects of combining AT1R antagonists with other anti‐inflammatory or neuroprotective agents to enhance therapeutic outcomes.

### 3.2 Oxidative Stress and Mitochondrial Dysfunction

Among the myriad molecular pathways implicated in addiction, ROS have emerged as key players in the regulation of neuroplasticity and memory processes. Specifically, NADPH oxidase (NOX), a major enzymatic source of ROS in the brain, has been shown to play a pivotal role in the reinforcement of addictive memories. NOX‐derived ROS are not merely toxic byproducts of cellular metabolism but are actively involved in redox signalling pathways that modulate synaptic strength, dendritic spine remodelling and LTP, a cellular correlate of memory formation [74]. The activation of NOX isoforms, particularly NOX2, in response to drug exposure is a critical event in the initiation and maintenance of addictive behaviours. This activation leads to the production of superoxide anions (O2•−), which are rapidly dismutated to hydrogen peroxide (H_2_O_2_), a more stable and diffusible ROS. These ROS species act as signalling molecules that enhance glutamatergic transmission, promote dendritic spine formation, and facilitate LTP, thereby strengthening synaptic connections associated with drug‐related cues and contexts [75]. Relevant studies have demonstrated that cocaine administration increases NOX2 expression and ROS levels in the NAc, which correlates with enhanced synaptic plasticity and the persistence of drug‐associated memories. Importantly, drug‐associated cues and contexts can reactivate NOX‐dependent ROS signalling, even in the absence of the drug itself, thereby reinforcing the salience and durability of addictive memories [76]. This reactivation process is thought to underlie the maladaptive learning that drives relapse, highlighting the important role of NOX‐derived ROS in the maintenance of addiction (Figure 4).

At the molecular level, NOX‐dependent ROS generation influences addictive memory through intricate interactions with downstream signalling cascades and epigenetic mechanisms. ROS modulate the activity of redox‐sensitive proteins, including PKC, ERK and NF‐κB, which are critical for synaptic plasticity and gene expression. ROS‐induced activation of ERK signalling enhances the transcription of immediate early genes such as c‐Fos and Zif268, which are essential for memory consolidation [77, 78]. ROS can directly modify synaptic proteins, including NMDA and AMPA receptors, thereby altering their function and contributing to the strengthening of drug‐associated synaptic connections [79]. NOX‐derived ROS have been implicated in the epigenetic regulation of addiction‐related genes through mechanisms such as DNA methylation and histone modification. These epigenetic changes can lead to long‐lasting alterations in gene expression that perpetuate addictive behaviours [80]. ROS‐mediated oxidation of DNA methyltransferases (DNMTs) and histone deacetylases (HDACs) can result in the dysregulation of genes involved in synaptic plasticity and reward processing, further entrenching the neural adaptations associated with addiction [81].

Pharmacological inhibition of NOX activity or genetic ablation of NOX isoforms has been shown to attenuate drug‐induced ROS production, reduce synaptic plasticity and impair the formation and maintenance of addictive memories. Administration of NOX inhibitors such as apocynin or genetic deletion of NOX2 in mice has been demonstrated to block cocaine‐induced increases in dendritic spine density and LTP in the NAc, as well as to reduce drug‐seeking behaviour in conditioned place preference (CPP) and self‐administration paradigms [82]. These findings underscore the importance of NOX‐derived ROS in the neuroplasticity underlying addiction and suggest that targeting this pathway could offer novel therapeutic strategies for the treatment of substance use disorders.

### 3.3 Epigenetic Modulation

One of the most intriguing mechanisms by which AT1R influences addiction‐related behaviours is through the regulation of histone modifications, particularly histone H3 lysine 9 acetylation (H3K9ac) [83]. H3K9ac is a well‐characterized epigenetic mark associated with transcriptional activation, and its dynamic regulation has been shown to play a pivotal role in the expression of genes involved in synaptic plasticity and addiction [84]. AT1R activation could enhance H3K9ac levels at the promoters of key addiction‐related genes, including ΔFosB and cAMP response element‐binding protein (CREB) [85, 86]. ΔFosB, a stable transcription factor induced by chronic drug exposure, is known to drive long‐term neuroadaptations that underlie addiction [87]. CREB, a critical mediator of neuronal plasticity, is essential for the consolidation of drug‐associated memories [80].

The molecular mechanisms governing AT1R‐mediated histone modifications are intricate and encompass a multitude of signalling pathways. Upon stimulation by Ang II, AT1R initiates G‐protein‐coupled signalling cascades, which include the activation of PKC and MAPKs. These pathways can directly or indirectly influence the activity of histone acetyltransferases (HATs) and histone deacetylases (HDACs), enzymes responsible for the addition and removal of acetyl groups on histones, respectively [88]. AT1R signalling has been shown to enhance the activity of CREB‐binding protein (CBP)/p300, a well‐known HAT that catalyses H3K9ac [89]. This epigenetic regulation by AT1R provides a mechanistic link between extracellular signalling and chromatin remodelling, highlighting its role as a critical modulator of addiction‐related gene expression.

In addition to its role in regulating ΔFosB and CREB, AT1R signalling may also influence other addiction‐related genes through similar epigenetic mechanisms. AT1R has been associated with the upregulation of brain‐derived neurotrophic factor (BDNF), a critical regulator of synaptic plasticity and reward processing, whose expression is known to be regulated by H3K9ac, and AT1R‐mediated increases in H3K9ac at the BDNF promoter could contribute to its enhanced transcription in response to drug exposure [90, 91]. Furthermore, AT1R signalling may interact with other epigenetic modifications, such as DNA methylation and histone methylation, to fine‐tune gene expression in addiction. Activation of the AT1R has been shown to reduce the expression of DNA methyltransferases (DNMTs), leading to decreased DNA methylation at specific gene promoters and consequently enhanced transcriptional activity [92]. The interplay between AT1R and histone modifications in the context of addiction is further highlighted by preclinical studies that demonstrate the therapeutic potential of modulating AT1R. Pharmacological inhibition of AT1R with losartan or genetic deletion of AT1R in animal models has been found to reduce drug‐seeking behaviours and attenuate the molecular changes associated with addiction [7, 93]. Importantly, these findings indicate that AT1R signalling functions as a pivotal epigenetic regulator, bridging the gap between extracellular signalling pathways and chromatin remodelling processes in the context of addiction.

## 4 Translational Medicine Perspective: Potential and Challenges of AT1R‐Targeted Therapy

### 4.1 Repositioning of Existing Drugs

Addiction remains a significant global public health challenge. Despite advances in understanding the neurobiological underpinnings of addiction, effective treatments are limited and relapse rates remain high [94]. Recent preclinical studies have highlighted the role of the RAS in modulating the brain's reward and stress pathways, which are critically involved in addiction [14]. ARBs, a class of medications commonly employed in the management of hypertension, have recently attracted considerable attention due to their potential to modulate neuroinflammation, oxidative stress and synaptic plasticity, which are processes that become dysregulated in addiction [95]. Clinical trials investigating ARBs in addiction have shown promising results across various substance use disorders. Studies on candesartan in alcohol use disorder reported reductions in withdrawal symptoms, such as anxiety and agitation, as well as improvements in mood and sleep quality [96]. These findings indicate that ARBs have the potential to attenuate neuroadaptive changes caused by chronic substance use, particularly the dysregulation of dopamine and glutamate signalling within the mesolimbic pathway. Furthermore, preclinical evidence suggests that ARBs can mitigate neuroinflammation and oxidative stress, both of which play critical roles in the progression of addiction and the development of comorbid psychiatric disorders such as depression and anxiety [97] (Table 2). By modulating both reward and stress pathways, ARBs present a distinctive therapeutic potential for addressing addiction.

**TABLE 2:** Some drugs and related pathways.

Drug name	Targeted pathways	Mechanisms	Potential relevance to addiction	References
Losartan	MAPK/ERK pathway TGF‐β/Smad pathway	Inhibits Ang II/AT1R axis, reduces ERK phosphorylation and TGF‐β‐mediated fibrosis	ERK pathway regulates synaptic plasticity and memory consolidation in addiction; suppression may reduce drug‐seeking behaviour	[98, 99]
Valsartan	NF‐κB pathway JAK/STAT pathway	Blocks AT1R, inhibits inflammatory cytokine release and STAT3 activation	NF‐κB promotes neuroinflammation and synaptic remodelling in addiction; inhibition may attenuate cocaine reward effects	[100]
Telmisartan	PPAR‐γ pathway PI3K/Akt pathway	Activates PPAR‐γ (improves metabolism); inhibits PI3K/Akt anti‐apoptotic signalling	PPAR‐γ activation may modulate dopaminergic systems; PI3K/Akt inhibition reduces morphine‐induced reward behaviour	[101, 102]
Irbesartan	Oxidative stress (ROS) TGF‐β pathway	Suppresses NADPH oxidase activity and ROS production; delays TGF‐β‐mediated fibrosis	ROS contributes to relapse mechanisms; ROS reduction may mitigate drug‐induced neuronal damage and craving	[103, 104]
Candesartan	NO/cGMP pathway ERK1/2 pathway	Enhances NO release; inhibits ERK1/2‐mediated cell proliferation	NO/cGMP pathway regulates synaptic plasticity in cocaine addiction; ERK suppression may weaken addiction memory	[105, 106]
Olmesartan	eNOS pathway NADPH oxidase pathway	Enhances eNOS activity (improves endothelial function); inhibits NADPH oxidase‐mediated oxidative damage	eNOS activity links to dopamine release; vascular modulation may indirectly influence addictive behaviours	[107]
Azilsartan	ACE2/Mas axis	Activates ACE2/Mas axis (counteracts Ang II)	ACE2/Mas axis may reduce neuroinflammation and oxidative stress, potentially impacting relapse	[108]

*Note:* Clinical trial number: not applicable.

Notwithstanding these promising results, a number of challenges remain to be addressed in order to fully harness the potential of ARBs for addiction treatment. First, the heterogeneity of addiction phenotypes, encompassing variations in genetic susceptibility and environmental influences, presents significant challenges to the design and interpretation of clinical trials [109]. Second, although ARBs are generally well‐tolerated among hypertensive populations, their safety and efficacy in non‐hypertensive individuals with addiction have not been extensively studied. Prolonged use of ARBs in this population may necessitate vigilant monitoring for potential adverse effects, including hypotension and renal dysfunction [110]. Mechanistic studies are crucial for elucidating the precise pathways through which ARBs exert their therapeutic effects. Investigating the role of AT1R in modulating synaptic plasticity and neurogenesis may offer valuable insights into the long‐term benefits of ARB treatment. The combination of ARBs with cognitive‐behavioural therapy or pharmacological agents such as naltrexone or bupropion may synergistically address multiple facets of addiction [111].

### 4.2 Design of New AT1R Antagonists

The therapeutic potential of AT1R antagonists has been constrained by their inadequate permeability across the BBB and insufficient selectivity, which may result in systemic adverse effects [112]. Recent efforts have focused on overcoming these challenges through innovative drug design strategies. One approach involves the structural modification of existing AT1R antagonists to enhance their lipophilicity while concurrently reducing molecular weight, thereby improving BBB. Additionally, prodrug strategies, where the active compound is masked by a lipophilic moiety that is cleaved upon BBB passage, have shown promise in enhancing CNS delivery [113]. Another promising approach involves the utilization of nanocarriers, such as liposomes or polymeric nanoparticles, which are capable of encapsulating AT1R antagonists and facilitating their transport across the BBB through receptor‐mediated transcytosis or passive diffusion [114]. These nanocarriers can be functionalized with targeting ligands to further enhance their specificity for the brain.

The BBB presents a formidable barrier to drug delivery, particularly for large or polar molecules. To address this, researchers have employed molecular modification strategies to optimize the physicochemical properties of AT1R antagonists. Recent studies have demonstrated that subtle modifications, such as the addition of a fluorine atom to the phenyl ring of losartan derivatives, can significantly enhance brain uptake while retaining high AT1R affinity [115]. Reducing molecular weight by removing non‐essential functional groups or optimizing the core scaffold can improve BBB permeability. The development of compact and rigid structures with minimal polar surface area has resulted in compounds that exhibit superior CNS penetration. These structural modifications not only enhance brain delivery but also significantly improve the pharmacokinetic profile, rendering them more appropriate for chronic administration in addiction therapy [116].

Selectivity is a critical consideration in the development of AT1R antagonists, as off‐target interactions with other angiotensin receptors, such as AT2R, can lead to adverse effects [117]. Computational modelling and high‐throughput screening have emerged as powerful tools for identifying selective ligands. Molecular docking studies and molecular dynamics simulations provide detailed insights into the binding interactions between AT1R and its antagonists, enabling the rational design of compounds with improved selectivity. Virtual screening of chemical libraries has identified novel scaffolds that exhibit high affinity for AT1R while minimizing interactions with AT2R. High‐throughput screening of large compound libraries has also yielded promising candidates with potent AT1R blockade and minimal off‐target activity [118]. These approaches have been complemented by pharmacophore modelling and quantitative structure–activity relationship (QSAR) analysis, which facilitate the optimization of lead compounds [119]. By integrating these computational and experimental techniques, researchers have developed AT1R antagonists with enhanced pharmacokinetic and pharmacodynamic properties, paving the way for more effective addiction therapies [120].

### 4.3 Combined Treatment Strategies

Methadone, a μ‐opioid receptor agonist, is widely used in opioid addiction maintenance therapy, while naltrexone, an opioid receptor antagonist, is employed to prevent relapse in both opioid and alcohol dependence [121, 122]. However, these treatments often exhibit limited efficacy due to the multifaceted nature of addiction, which involves dysregulation of multiple neurotransmitter systems and neuroadaptive changes in brain circuits [123]. Recent studies have highlighted the potential of AT1R antagonists, commonly used as antihypertensive agents, to augment the therapeutic effects of existing addiction treatments [50]. AT1R antagonists have been shown to modulate dopaminergic signalling, reduce neuroinflammation and mitigate stress‐related behaviours, all of which are implicated in the pathophysiology of addiction [14]. By targeting the RAS in the brain, AT1R antagonists may complement the mechanisms of methadone and naltrexone, offering a novel approach to enhance treatment outcomes.

Methadone and naltrexone primarily modulate the opioid system, but addiction also involves dysregulation of the mesolimbic dopamine system, glutamatergic signalling and stress responses mediated by the HPA axis [124]. AT1R antagonists have been demonstrated to reduce dopamine release in the nucleus accumbens, a critical brain region implicated in reward processing. This reduction consequently diminishes the reinforcing effects of addictive substances [125]. Additionally, AT1R antagonists can mitigate the neuroinflammatory response associated with chronic drug use, which contributes to neuronal damage and cognitive deficits [126]. These agents have been reported to reduce anxiety‐like behaviours and stress‐induced drug‐seeking by modulating CRF signalling in the amygdala [127]. When combined with methadone or naltrexone, AT1R antagonists may thus provide a more comprehensive therapeutic strategy by targeting both the reward and stress pathways, potentially reducing relapse rates and improving long‐term recovery. Future clinical trials are essential to investigate the safety and efficacy of combining AT1R antagonists with current therapeutic regimens. This approach shows potential in addressing unmet needs in addiction treatment.

## 5 Future Directions

### 5.1 Gender and Individual Differences

Although addiction affects both males and females, there are notable sex differences in susceptibility, progression and treatment outcomes [128]. Females, for instance, often exhibit faster progression from initial drug use to addiction, greater withdrawal severity and higher rates of relapse compared with males [129]. These disparities suggest that biological and hormonal factors play a critical role in modulating addiction vulnerability. AT1R signalling is involved in regulating dopamine release, synaptic plasticity and stress responses—processes that are central to the development and maintenance of addictive behaviours [130]. Importantly, emerging evidence indicates that AT1R expression and function are differentially regulated in males and females, providing a potential mechanism for the observed sex differences in addiction susceptibility [131]. Previous research has found that AT1R expression is higher in females in key brain regions associated with reward processing, such as NAc and PFC [132]. This elevated AT1R activity in females may potentiate dopamine release in response to drugs of abuse, thereby reinforcing drug‐related behaviours and heightening vulnerability to addiction. For example, in animal models of nicotine addiction, female rodents exhibit greater AT1R‐mediated dopamine release in the NAc compared with males, which correlates with increased drug self‐administration and faster acquisition of addictive behaviours [133]. Hormonal fluctuations, particularly oestrogen, have been shown to modulate AT1R signalling [132]. Together, these findings suggest that sex‐specific regulation of AT1R signalling plays a critical role in shaping addiction vulnerability, with females being particularly susceptible due to higher AT1R activity and hormonal influences.

The interplay between AT1R signalling and stress pathways further underscores the importance of sex‐specific mechanisms in addiction. Chronic stress is a well‐established risk factor for addiction, as it dysregulates the brain's reward system and increases vulnerability to drug use [134]. AT1R signalling is a key mediator of stress‐induced changes in the brain, as it enhances dopamine release and sensitizes the reward circuitry to drugs of abuse [19]. However, the mechanisms by which stress modulates AT1R signalling appear to differ between males and females [135]. In males, stress‐induced AT1R activation is primarily mediated by glucocorticoid signalling, which upregulates AT1R expression in the NAc and PFC. In contrast, in females, oestrogen appears to play a more prominent role in stress‐induced AT1R activation, potentially explaining their greater vulnerability to stress‐related addiction [136, 137]. For example, female rodents subjected to chronic stress demonstrate elevated AT1R expression and enhanced dopamine release in response to drug stimuli compared with their male counterparts. This phenomenon is associated with increased drug‐seeking behaviour and a higher likelihood of relapse [138]. These sex‐specific stress‐AT1R interactions may contribute to the faster progression from recreational drug use to addiction observed in females, as well as their heightened susceptibility to stress‐induced relapse.

Genetic factors play a significant role in contributing to sex‐specific differences in AT1R signalling and susceptibility to addiction. Polymorphisms in the AT1R gene have been associated with increased risk of addiction, with some variants showing sex‐specific effects [139]. Epigenetic modifications, such as DNA methylation and histone acetylation, have been shown to regulate AT1R expression in a sex‐specific manner [132]. These epigenetic changes may be influenced by environmental factors, such as stress and drug exposure, further contributing to the sex differences in addiction susceptibility [140]. Elucidating the genetic and epigenetic regulation of AT1R signalling in males and females may offer significant insights into the mechanisms that underlie sex‐specific susceptibility to addiction.

### 5.2 Multi‐Omics Integration Studies

The advent of single‐cell RNA sequencing (scRNA‐seq) and spatial transcriptomics has revolutionized our understanding of cellular heterogeneity and the spatial organization of tissues, particularly in the context of the brain. These technologies have enabled researchers to dissect the molecular profiles of individual neurons and glial cells, revealing previously unappreciated diversity within neuronal subpopulations [141]. In the context of addiction, AT1R expression has been dynamically regulated in dopaminergic neurons of VTA and their projections to NAc, key regions in the brain's reward circuitry [50]. These findings suggest that AT1R signalling may be a potential therapeutic target for modulating reward‐related behaviours and addiction.

Spatial transcriptomics has further complemented these insights by providing a spatial context to the expression patterns of AT1R within brain tissue. This approach was able to reveal that AT1R expression is not evenly distributed, but is confined to specific layers or subregions within brain regions, such as PFC, hippocampus and amygdala [142]. For example, in the PFC, AT1R is predominantly expressed in deep‐layer pyramidal neurons, which are known to play a crucial role in executive functions and decision‐making processes [143]. The spatial resolution of AT1R expression has also been linked to its functional role in stress‐induced plasticity, where its activation in specific neuronal subpopulations can lead to long‐term changes in synaptic strength and connectivity [144]. The dynamic changes are often accompanied by alterations in the expression of downstream signalling molecules, suggesting a complex regulatory network that fine‐tunes AT1R activity in a context‐dependent manner. Collectively, these findings underscore the importance of AT1R in specific neuronal subpopulations and its potential as a therapeutic target for neuropsychiatric disorders, including addiction and depression.

### 5.3 Difficulty With Clinical Translation

Determining the optimal dosage for pharmacological interventions in addiction treatment remains a significant challenge, as individual variability in drug metabolism, genetic predisposition and the severity of addiction can greatly influence therapeutic outcomes. Many drugs targeting addiction‐related pathways, such as those modulating AT1R or other neurotransmitter systems, exhibit a narrow therapeutic window, where suboptimal doses may fail to elicit a therapeutic effect, whereas excessive doses can lead to adverse side effects or even exacerbate addictive behaviours [145]. For instance, medications like naltrexone or buprenorphine, which are used to treat opioid use disorder, require careful titration to balance efficacy with tolerability [146, 147]. The dynamic nature of addiction—characterized by fluctuating levels of craving, withdrawal and relapse—further complicates dosage optimization. Personalized medicine approaches, including pharmacogenomics and real‐time monitoring of drug levels, are being explored to tailor dosages to individual patients. However, these strategies are still in their infancy and face practical challenges, such as cost, accessibility and the need for robust biomarkers to guide treatment decisions. Additionally, the potential for drug interactions, particularly in individuals with polysubstance use, adds another layer of complexity to dosage determination, necessitating a comprehensive understanding of each patient's unique pharmacological profile.

Long‐term safety and the prevention of relapse are equally critical challenges in addiction treatment. Many pharmacological agents used to manage addiction, such as methadone or antipsychotics, are associated with significant side effects when used over extended periods, including metabolic disturbances, cardiovascular risks and cognitive impairments [148]. These risks are particularly concerning given the chronic nature of addiction, which often requires prolonged or even lifelong treatment. Furthermore, the neuroadaptive changes that occur in the brain during addiction, such as alterations in reward circuitry and stress responses, can persist long after drug cessation, creating a vulnerability to relapse [31]. Despite advances in understanding the neurobiology of addiction, relapse rates remain high, underscoring the need for more effective strategies to sustain recovery. Emerging approaches, such as the use of long‐acting injectable formulations or implantable devices, aim to improve adherence and reduce the risk of relapse by providing sustained drug delivery [149]. However, these innovations must be carefully evaluated for their long‐term safety and potential to induce dependence or other adverse effects. Behavioural interventions, such as cognitive‐behavioural therapy and contingency management, are often used in conjunction with pharmacological treatments to address the psychological and social factors contributing to relapse [150]. Nevertheless, integrating these multimodal approaches into a cohesive treatment plan remains a challenge, particularly in resource‐limited settings. Ultimately, overcoming these challenges will require a multidisciplinary effort, combining advances in pharmacology, neuroscience and behavioural science to develop safer, more effective and personalized treatments for addiction.

## 6 Conclusion

The AT1R, a key component of the RAS, has emerged as a critical regulatory node in the CNS, with significant implications for neuropsychiatric disorders, including addiction. Beyond its traditional role in cardiovascular regulation, AT1R is widely expressed in brain regions involved in reward processing, stress response and cognitive control, such as the mesolimbic dopamine system, prefrontal cortex and amygdala. AT1R activation modulates neurotransmitter systems (e.g., dopamine, glutamate and GABA), synaptic plasticity, neuroinflammation and oxidative stress—processes dysregulated in addiction. Beyond addiction, AT1R modulation shows promise in depression, anxiety, cognitive impairment and neurodegenerative diseases. AT1R antagonists ameliorate depressive‐like behaviours, reduce anxiety and improve cognitive function in preclinical models, highlighting its broad therapeutic potential. However, challenges remain, including limited BBB penetrance of current AT1R antagonists and the heterogeneity of AT1R signalling across brain regions and cell types. Advances in drug delivery systems, single‐cell omics and spatial transcriptomics are needed to optimize targeted interventions. Realizing AT1R's full therapeutic potential requires multidisciplinary collaboration, large‐scale clinical trials and public–private partnerships to accelerate translational research. By addressing these challenges, AT1R‐targeted therapies could transform the treatment landscape for addiction and other CNS disorders, offering a framework for precision neuropsychiatry.

## Funding

This work was supported by the National Natural Science Foundation of China (82301680: Z.S.), the Young Doctor Innovation Research Program of Ningbo (2024J469: Z.S.), Ningbo Public Welfare Research Program Project (2022S075: Y.L.), Li Dasan and Ye Yaozhen Couple Li Benjun Marine Biomedicine R&D Fund of Ningbo University (2025‐02), and Ningbo Yongjiang 2035 Research Project (2024Z190).

## Data Availability

Data sharing not applicable to this article as no datasets were generated or analysed during the current study.
